# miRNAs and Leukotrienes in Respiratory Syncytial Virus Infection

**DOI:** 10.3389/fped.2021.602195

**Published:** 2021-04-29

**Authors:** Zhi Liu, Panpan Fan, Ming Chen, Yueshi Xu, Dongchi Zhao

**Affiliations:** ^1^Department of Pediatrics, Children's Digital Health and Data Center, Zhongnan Hospital of Wuhan University, Wuhan, China; ^2^Xiangyang Central Hospital, Affiliated Hospital of Hubei University of Arts and Science, Xiangyang, China

**Keywords:** microRNA, arachidonic acid, leukotriene, respiratory syncytial virus, children

## Abstract

MicroRNAs (miRNAs) are small, non-coding RNAs that regulate posttranscription by binding to 3′-untranslated regions of target mRNAs. Recent functional studies have elucidated mechanisms that miRNAs regulate leukotriene synthesis by perturbing arachidonic acid metabolism. Both microarrays and high-throughput sequencing revealed distinct differential expression of miRNAs in children with respiratory syncytial virus (RSV) infection compared with healthy controls. Abnormal miRNA expression may contribute to higher leukotriene levels, which is associated with airway hyperreactivity. Targeting miRNAs may benefit to restore the homeostasis of inflammatory reaction and provide new strategies to alleviate airway hyperreactivity induced by RSV. In this article, we provide an overview of the current knowledge about miRNAs modulating leukotrienes through regulation of arachidonic acid metabolism with a special focus on miRNAs aberrantly expressed in children with RSV infection.

## Introduction

Respiratory syncytial virus (RSV) is the most common pathogen of acute lower respiratory infection in children and the leading cause of hospitalization in childhood, which results in a great burden on global health-care services (1, 2). Due to high morbidity and mortality, RSV infection poses a serious threat to children's health, especially for premature infants, or infants with congenital heart disease or primary immunodeficiency (3–5). RSV is a single-strand negative RNA virus belonging to the Paramyxoviridae family and the Pneumovirus genus. RSV encodes 11 proteins including two non-structural proteins 1 and 2 (NS1 and NS2), structural proteins such as membrane envelope glycoproteins (F and G), and matrix proteins (M). These proteins are critical pathogenic factors to induce airway hyperreactivity (AHR), including immune disorder, overexpression of Th2-type cytokines, and inflammatory disequilibrium (6–8). RSV infection in early childhood induces AHR and contributes to the subsequent development of recurrent wheezing (9, 10). Increasing leukotriene levels are crucial for the occurrence of AHR after RSV infection and related to recurrent wheezing attacks (11). However, the mechanism of leukotriene upregulation after RSV infection is not clear yet.

MicroRNAs (miRNAs), a type of endogenous non-coding RNAs with a length of 18 to 25 nucleotides, are the most important molecules in the posttranscriptional regulation of gene expression (12). MiRNAs nearly precisely fine-tune the intensity of the cellular signals which are activated by RSV and associated with AHR (13–16). Many miRNAs have been confirmed to be abnormally expressed after RSV infection, some of which negatively regulate AHR, such as miR-24, miR-27, and let-7 family (17–21), others positively, such as miR-140-5p and miR-146b (22, 23). Recently, accumulating evidence demonstrated that miRNAs play an important role in regulating the synthesis and balance of lipid inflammatory mediators (24). The regulatory networks of miRNAs on leukotriene synthesis after RSV infection have not been explained in detail. Therefore, here we present an updated review on this issue.

## Leukotriene Synthesis and RSV Infection

**Figure 1:**
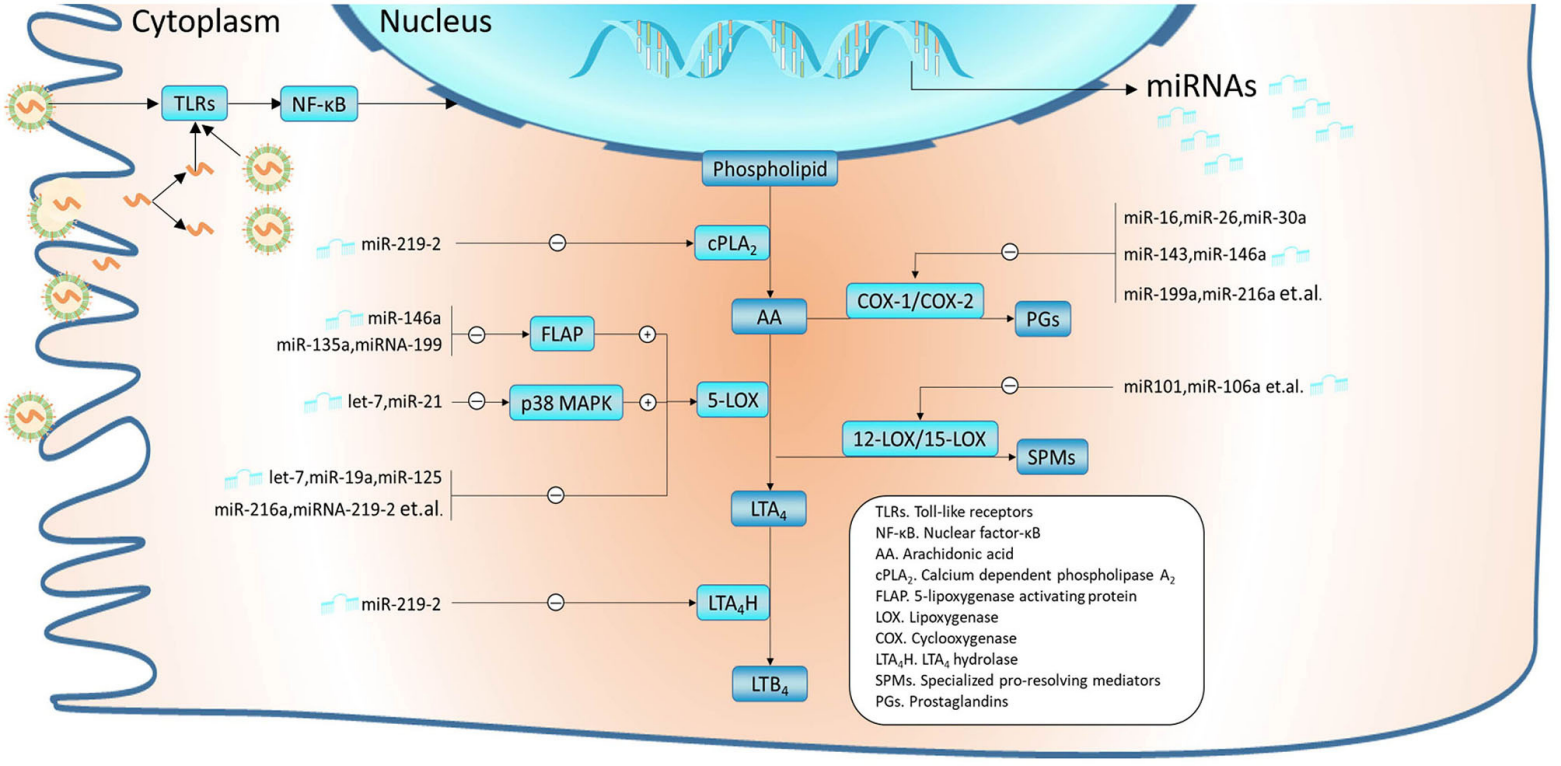
miRNAs were validated to modulate the metabolism of arachidonic acid (AA). In airway epithelial cells, RSVs are recognized by PAMPs such as TLRs, which subsequently activate signal pathways like NF-κB and affect the expression profile of miRNAs. MiRNAs regulate leukotriene synthesis by modulating AA metabolism, which mainly includes the LOX pathway and COX pathway. Thereinto, miR-219-2 can inhibit several enzymes such as cPLA2, 5-LOX, and LTA_4_H. Some miRNAs have been confirmed to inhibit the expression of 5-LOX, such as let-7, miR-19a, and miR-125. In addition, miR-146a, miR-135a, and miR-199 can regulate 5-LOX enzyme activity by downregulating FLAP; let-7 and miR-21 by downregulating p38 MAPK. Interference with these miRNAs may contribute to the consistently overexpressed leukotrienes. MiRNAs such as miR-16, miR-26, miR-30a, miR-143, and miR-146a may lead to the conversion of more AA to leukotriene by inhibiting the COX pathway. Besides, overexpression of miRNAs like miR-101 and miR-106a can suppress 12-LOX/15-LOX, which could disrupt the balance between pro-inflammatory and inflammation-resolving mediators. Among these miRNAs, let-7, miR-16-5p, miR-19a, miR-21, miR-26b, miR-30a-5p, miR-125a, miR-143, and miR-146a have been found abnormal expression in RSV infection.

In humans, leukotrienes are produced by leukocytes, bronchial epithelial cells, and fibroblasts. The biosynthetic pathway of leukotrienes is briefly outlined in Figure 1. 5-Lipoxygenase (5-LOX) and 5-lipoxygenase-activating protein (FLAP) are critical determinants of leukotriene biosynthesis (25). 5-LOX carries out the first steps in the 5-LOX pathway of leukotriene synthesis. FLAP plays an important role in the coupling of cPLA2 to 5-LOX at the perinuclear membrane. Under the action of 5-LOX and FLAP, leukotriene A_4_ (LTA_4_) is synthesized from free arachidonic acid (AA) released by phospholipase A_2_ (PLA_2_) from membrane glycerophospholipids (26). Then, LTA_4_ is transformed to LTB_4_ and LTC_4_ by LTA_4_ hydrolase (LTA_4_H) and LTC_4_ synthase (LTC_4_S), respectively. LTD_4_ and LTE_4_ are synthesized from LTC_4_. LTC_4_, LTD_4_, and LTE_4_ are collectively called cysteinyl leukotrienes (CysLTs) (27). Released AA can also be oxidized to prostaglandin H2 (PGH_2_) by cyclooxygenase (COX) or converted to specialized pro-resolving mediators (SPMs) by 12-lipoxygenase (12-LOX)/15-lipoxygenase (15-LOX). Inhibition or enhancement of these metabolic pathways of AA may also take a great effect on the formation or resolving of leukotrienes. Furthermore, all these enzymes mentioned above play essential regulatory roles and do not act alone but form distinct complexes on the nuclear membrane (28).

Over the last decades, different studies have demonstrated the elevations in locally produced LTC_4_ during RSV infection (29, 30), which may persist beyond 1 month after the onset of infection (31). The level of LTC4 was positively correlated with disease severity (30, 32). Persistent elevated LTC_4_ levels in nasopharyngeal aspirates (NPAs) were observed in infants who suffered from wheezing compared with the group without symptoms after the acute RSV infection (33). Besides, high levels of LTB_4_ and CysLTs were detected in the culture supernatant of RSV-infected human bronchial epithelial cells (HBEC) (34, 35). Further studies suggest that RSV induces the expression of 5-LOX in bronchial epithelial cells and thus increases airway inflammation (35). In a RSV-infected mouse model, inhibiting FLAP could reduce inflammation (36). In addition, RSV may regulate leukotriene synthesis by disrupting the COX pathway. The expression levels of COX-2 are upregulated in peritoneal and alveolar macrophages of cotton rats infected with RSV (37).

## miRNAs Expression Profile Changed in RSV Infection

MiRNAs have been intensively studied in the last decades since its discovery in Caenorhabditis elegans in 1993 (38). Whereas the majority of miRNAs have their own gene loci, there are 30% of miRNAs co-transcribed from the introns of protein-coding host genes (39, 40). Recent studies have uncovered that miRNA biogenesis can be regulated at multiple levels, including transcription, processing, modification by RNA editing, Argonaute loading, and RNA decay (41). There is complicated cross talk between miRNA synthesis and other cellular signaling pathways (42, 43). RSV could affect the biogenesis of miRNAs through G, NS1, and NS2 proteins by affecting cellular signal transduction (17, 44). RSV G proteins can upregulate host miRNA (let-7f, miR-24) expression to weaken the host antivirus response by inhibiting the formation of interferon-λ (IFN-λ) (45). In NHBE cells, the promoting role of NS1 and NS2 proteins in the expression of let-7i and miR-30b is mediated through interferon-β (IFN-β) and nuclear factor-κB (NF-κB) signaling separately (46). Similarly, RSV NS1 modifies miR-24 expression via transforming growth factor-β (TGF-β) in A549 cells (47). It should be noted that RSV has cell-specific regulation of miRNA expression. Let-7b can be upregulated in dendritic cells (DCs), while the upregulation of let-7i and miR-30b requires viral replication (46).

RSV could significantly change the expression profile of miRNA in human bronchial epithelial cells, of which 24 miRNAs were greatly downregulated and two miRNAs were upregulated (48). In clinical samples and *in vitro* cell experiments, miRNA expression showed a distinct profile. The regulation of RSV on miRNAs was first observed in A549 cells, including elevated levels of let-7a, let-7f, miR-24, miR-36, miR-520, and miR-337 and decreased levels of miR-198, miR-224, and miR-595 (49). Recently, miR-29 has been identified to be upregulated by RSV NS1 protein not only in A549 cells but also in NPAs (50). Apart from A549 cells, normal epithelial cells and Hep-2 cells are the most common models of RSV infection. In RSV persistently infected Hep-2 cells, miRNA-146-5p, miR-let-7c-5p, miR-221, and miR-345-5p are differentially expressed (51). In addition to the cellular level, RSV infection also leads to changes in the expression of exosome miRNAs such as Let-7a, Let-7f, miR-320a, miR-21, miR-4449, and miR-22 (52). MiRNAs in exosome, which is an important tool for intercellular communication, play an important role in pathogenesis and protection against diseases. In this way, the change of miRNA expression by RSV infection can not only act on the infected cells themselves but also regulate intercellular communication, thus affecting the local microenvironment.

Abnormal miRNA profiles have also been verified in clinical specimens such as nasal epithelium cytology brushings and peripheral blood from infants infected by RSV. Our former research found a significant alternation of miRNA expression profile in the peripheral blood of infants after RSV infection (53). The upregulated miRNAs include miR-106b-5p, miR-181a-5p, miR-20b-5p, miR-342-3p, and miR-652-3p, while the downregulated including miR-122-5p, miR-320e, miR-320d, miR-877-5p, miR-92b-5p, and let-7c-5p. Considering the different expression levels of miR-125a and miR-429 in NPAs of children with different severity of RSV infection (21), miRNAs may become potential biomarkers for the diagnosis and treatment of RSV infection. Furthermore, an increasing number of miRNAs (Figure 1) are confirmed to directly regulate leukotriene levels by targeting proteins associated with AA metabolism (24, 54, 55). The majority of miRNAs mentioned above were involved in pathways related to the immune and inflammatory responses such as macrophage polarization states (56–58), which is closely associated with the balance between leukotriene synthesis and regression and the following severe airway inflammation (59–61).

## The Regulatory Functions of miRNAs Related to Leukotriene Synthesis on the Inflammatory Response During RSV Infection

Many miRNAs are directly or indirectly related to leukotriene synthesis, however, only a few of them have been confirmed to be significantly changed and involved in the regulation of leukotriene synthesis in RSV infection. We matched the function in arachidonic acid metabolism and expression during RSV infection of these miRNAs (miR-125a, miR-19a, let-7, miR-146a, miR-30a-5p, miR-16-5p, miR-26b, miR-21, miR-143) together and summarized in Table 1. Next, we focus on the expression and role of several kinds of miRNA which are widely involved in the regulation of inflammatory pathways after RSV infection.

**Table 1:** Summary—miRNAs influencing the AA metabolism and abnormal expressed in RSV infection.

**miRNA**	**miRNAs and AA metabolism**				**RSV infection and miRNAs**			
	**Target gene**	**Cell type**	**Reference**	**Year**	**Expression level**	**Method**	**Sample source**	**Reference**	**Year**
hsa-miR-125b	5-LOX	Monocytes (MM6 cells), T-lymphocytes	(62)	(2015)	↓	miRNA microarray, qPCR	nasal mucosal specimens	(21)	(2015)
hsa-miR-19a	5-LOX	Monocytes (MM6 cells), T-lymphocytes	(62)	(2015)	↑	miRNA microarray	nasal mucosal specimens	(21)	(2015)
let-7	5-LOX	Endothelial cells (ECs) of mouse model	(63)	(2017)	↑	miRNA microarray	peripheral blood	(53)	(2017)
hsa-miR-146a	FLAP,COX-2	Lung cancer	(64, 65)	(2014) (2018)	↑	qPCR	Hep2	(51)	(2018)
hsa-miR-30a-5p	COX-2	Gastric cancer	(66)	(2017)	↑	NGS, qPCR	moDCs from human PBMCs	(67)	(2018)
hsa-miR-16-5p	COX-2	Cervical cancer, Hepatocellular carcinoma	(55, 68)	(2005) (2012)	↑	miRNA, microarray, qPCR	nasal mucosal specimens	(21)	(2015)
hsa-miR-26b	COX-2	Nasopharyngeal epithelial cancer	(69)	(2010)	↑	miRNA, microarray, qPCR	PBMCs	(49)	(2012)
hsa-miR-143	COX-2	Amnion mesenchymal cells	(70)	(2011)	↓	multiplex qPCR array	NHBEs	(48)	(2012)
hsa-miR-21	15-PGDH	Cholangiocarcinom	(71)	(2014)	↑	NGS, qPCR	exosomes derived from RSV-infected A549/SAE cells	(72)	(2012)

### MiR-19a

MiR-19a is a member of the miR-17-92 cluster which contains 6 miRNAs (miR-17, miR-18a, miR-19a, miR-19b, miR-20a, and miR-92) and is a potential regulator of several proliferation-related genes. MiR-19a is overexpressed in both asthma cases and RSV-infected patients. In asthma, miR-19a can promote the production of Th2 cytokine IL-13 by directly targeting PTEN, a signal transduction inhibitor suppressor of cytokine signaling 1 (SOCS1), and deubiquitinase A20 (73, 74). Similarly with leukotriene, IL-13 is a key driver of airway inflammation, inducing epithelial cell proliferation and mucus production, airway hyperreactivity, and eosinophil recruitment. RSV infection stimulates group 2 innate lymphoid cells (ILC2) to express a higher level of IL-13 through the thymic stromal lymphatic hormone in the mouse model (75). Interestingly, CysLTs induce ILC2 cell migration and promote the production of IL-13, and IL-13 increased bronchial smooth muscle cell (BSMC) CysLT1R protein expression in effect related to its concentration in *in vitro* experiments (76, 77).

In a prior study, miR-17-92 controls the proliferation and survival of CD8 T-cells by suppressing the expression of the phosphatase and tensin homolog (PTEN) (78). The decreasing formation of PTEN leads to the activation of the PI3K–Akt–mTOR signaling pathway, which causes memory differentiation inhibition (79). Moreover, both fatty acid synthesis and fatty acid uptake are stimulated in response to mTOR signaling, including polyunsaturated fatty acids, which are the immediate precursors of many lipids (80). In the NPAs of infants infected with RSV, the results of microarray support the upregulation of miR-19a-3p in the severe disease subgroup (21). We previously found that miR-106b-5p, a paralog of the miR-17-92 cluster family, was significantly increased in the peripheral blood of infants with RSV infection (53). Consistent with the function of miR-19a, miR-106b regulates the PI3K-Akt pathway by suppressing PTEN (81). MiRNA-19a and miR-106b may play an activator role in leukotriene synthesis.

### miR-125a

Also in the NPAs of infants infected with RSV, the expression of miR-125a in the mild and moderate disease subgroups was downregulated, while it was not expressed in the severe disease subgroup (21). Previous studies have promoted that miR-125a and miR-125b constitutively activate the NF-κB pathway by targeting the tumor necrosis factor alpha-induced protein 3, and miR-125a may participate in the self-regulatory loop of miR-125b and NF-κB (82). Prior research substantiates the belief that the NF-κB pathway plays a central role in mediating airway inflammation induced by RSV, and RSV can regulate miRNAs by the NF-κB pathway (46, 83).

### miR-146a

The expression of miR-146a is significantly altered by RSV infection, which could also be mediated by the activation of the NF-κB pathway (51, 84). The academic community has extensively explored the anti-inflammatory functions of miR-146a in the airway. Pro-inflammatory cytokines such as IL-1β, TNF-α, and IFN-γ can induce the expression of miR-146a in human airway smooth muscle cells (85). MiR-146a can negatively regulate inflammatory gene levels in numerous cell types, including monocytes, fibroblasts, and endothelial, airway smooth muscle, and epithelial cells (85–87). Based on these studies, RSV infection downregulates the expression of miR-146a which may play a key role in impairing inhibitory effects on inflammatory pathways such as leukotriene synthesis. For example, miR-146a enhances M2 macrophage polarization by activating peroxisome proliferator-activated receptors γ (88), while it negatively regulates TLR4 signaling which plays an essential role in the regulation of M1 macrophage polarization (89).

### Let-7 Family

Let-7 family miRNAs play an important role in inhibiting host innate immunity and promoting replication during RSV infection (17, 45, 52). RSV induces let-7 family miRNA levels. We have previously found that let-7c was increased by RSV in A549 cells and peripheral blood of infants (53). RSV may enhance nuclear transcription factors associated with let-7 synthesis by activating MAPK pathways through TLR signaling (90–92). Ras-ERK/MAPK signaling is repressed by let-7 miRNAs in humans and other species (92). This phenomenon may be a conserved regulatory mechanism. The overexpression of let-7 miRNAs may be one of the negative feedback loops for regulating MAPK. Furthermore, MAPK is associated with 5-LOX enzyme activity. p38 MAPK can be rapidly activated by RSV. Activated p38 phosphorylates and stimulates downstream kinase to phosphorylate 5-lipoxygenase. RSV is a potent inducer of NF-kB and p38 MAPK phosphorylation in A549 cells (90, 93). RSV induces high-mobility group box 1 (HMGB1) to release from human airway epithelial cells via NF-kB and TLR4 signaling pathways. Then, HMGB1 activates p38 MAPK and triggers the release of pro-inflammatory mediators (94). In an ovalbumin-sensitized murine model of asthma, let-7 miRNA downregulated IL-13 and relieved allergic airway inflammation (20).

### miR-21

MiR-21, which can be secreted by exosomes, is one of the most highly expressed members of the small non-coding miRNA family in many cell types and tissues. It is accepted as an activator of regeneration processes in tissue damage repair and tumor growth (63). In addition, miR-21 may be a common biomarker of inflammation-related diseases (95). Induced by many pro-inflammatory stimuli including pathogen-associated molecular patterns (PAMP) and danger-associated molecular patterns (DAMP), miR-21 subsequently triggers the inflammatory circuit and promotes the function of the immune system. It may be a negative regulation of the inflammatory process and an important switch for dispelling inflammation (96, 97). In RSV-infected cells, there was a significant upregulation in the composition of exosome miR-21(72, 98). Exosomes released from virus-infected A549 cells can alter innate immune responses through the induction of pro-inflammatory mediators. Antagonistic miR-21 treatment can inhibit eosinophil inflammation and AHR in RSV-induced steroid-insensitive mouse airway allergic disease models (99). Therefore, miR-21 may be a key signal to regulate the balance and transition between pro-inflammatory and immune activation. The regulatory roles of miR-21 on the synthesis of leukotrienes are described below.

### miR-26b and miR-16

These miRNAs are widely involved in the inflammation reaction induced by RSV infection. Microarray and NGS of RSV infection specimens have shown an obvious change in expression profile (21, 67). Peripheral blood mononuclear cells (PBMCs) in children with RSV infection had higher miR-26b levels, while miR-26b induced downregulation of the TLR4 signal *in vitro* (100). Similarly, RSV induced miR-26 in A549 cells (49). Besides, miR-26a is correlated with hypertrophic human airway smooth muscle cells, which is one of the hallmarks of airway remodeling in severe asthma (101). In a study aiming to establish whether miRNAs could be used to characterize or subtype asthmatic patients, circulating miR-16 was one of the most predictive of allergic and asthmatic status (102). Likewise, miR-16 upregulated by RSV may participate in the formation of AHR.

## Linkages Between miRNAs and Leukotrienes During Other Respiratory Virus Infections

Leukotrienes are also believed to contribute to the pathophysiology of respiratory infection by other viruses such as influenza, rhinovirus (RV), metapneumovirus (HMPV), and adenovirus. The sporadic association between leukotrienes and miRNAs has been reported during infection of these viruses. We included related studies in Table 2. Compared to RSV, influenza may increase leukotriene concentrations by inducing the 5-LOX pathway (103). Similar to RSV, the miRNA expression profile of host cells is significantly changed by influenza (111). Among these miRNAs, let-7, miR-21, and miR-29 have connections with AA metabolism and miR-29 activates COX-2 through epigenetic changes during influenza A infection (104). Alveolar lavage fluid of RV infection patients contains higher cysLT levels than the control group (112). This may be associated with the induction of 5-LOX, FLAP, and COX-2 (105). However, there are few reporters about leukotriene synthesis-related miRNAs except RV-increasing airway secretory miR-155 in young children (106). MiR-155 is associated with prostaglandin metabolism in cancer, but its roles in leukotriene synthesis have not been investigated yet. As to HMPV, which shows common symptoms of wheezing like RSV, one study has shown that bronchiolitis children infected with HMPV have higher leukotriene levels in blood and urine than the control group (107). However, the roles of 5-LOX and COX-2 in HMPV infection are still unknown. Nonetheless, montelukast, a selective CysLT1R antagonist, has been used to treat HMPV infection of hospitalized young children (113). A high-throughput sequencing study of HMPV-infected A549 cells shows upregulation of let-7f (108). Both upregulation of leukotrienes and let-7f in HMPV infection are consistent with RSV infection. This remains to be established. Unlike these RNA viruses, adenovirus reduces the release of arachidonic acid by inhibiting the translocation of cPLA2 to membranes (109). MiRNA (include miR-125, miR-19a, miR-191) levels in adenovirus-infected cells fluctuate in distinct stages (110). Whether or not miRNAs changed by these viruses are involved with leukotriene synthesis during infection still needs further investigations.

**Table 2:** miRNAs influencing the AA metabolism in other respiratory virus infection.

**Virus**	**Type**	**Virus & LTs**					**Virus & miRNAs**					
		**LTs levels**	**Enzymes levels**	**Sample source**	**Reference**	**Year**	**miRNAs**	**Expression level**	**Method**	**Sample source**	**Reference**	**Year**
Influenza	RNA	↑	5-LOX↑	Nasopharyngeal swabs and lavages	(103)	(2013)	miR-29	↓	qPCR	PBMC A549 cells	(104)	(2012)
RV	RNA	↑	5-LOX↑ FLAP↑ COX-2↑	BAL fluid	(105)	(2002)	miR-155	↑	miRNA microarray	Nasal airway secretions	(106)	(2016)
HMPV	RNA	↑	/	Serum and urine	(107)	(2019)	let-7	↑	qPCR	A549 cells	(108)	(2014)
Adenovirus	DNA	↓	cPLA_2_↑	A549 cells	(109)	(1997)	let-7 miR-125 miR-19a	↑/↓ ↑ ↓	miRNA microarray	human lung fibroblast	(110)	(2015)

## miRNAs Regulating the Synthesis of Leukotrienes

### miRNAs and Lipoxygenase Pathway

#### Expression and Activity of 5-LOX

During RSV infection, a damaged or inflamed bronchial epithelium synthesizes a higher level of leukotrienes by inducing 5-LOX (114), the most critical enzyme of leukotriene synthesis. Recent studies have demonstrated various types of miRNAs involved in the regulation of 5-LOX. For example, miR-219-2 can directly interact with the 3′ untranslated region (3′-UTR) of 5-LOX to downregulate the expression of 5-LOX mRNA in macrophages (115). Similarly, overexpression of miR-216a-3p in human colorectal cancer cell lines can directly bind to the 3′-UTR, causing the same effect on 5-LOX (116). MiR-19a-3p and miR-125-5p, which are abnormally expressed in RSV infection (21), have also been identified to directly regulate the expression of 5-LOX protein without affecting 5-LOX mRNA in monocyte line MM6 induced *in vitro* (62). MiR-674-5p can attenuate concanavalin A-induced liver injury in mice by downregulating 5-LOX (117). In rats with focal cerebral ischemia and reperfusion, miRNA-193b-3p can alleviate the injury by inhibiting the expression of 5-LOX (118). In a deficient mouse model, a decrease of let-7 miRNAs led to the upregulation of 5-LOX and subsequent aberrant activation of the leukotriene biosynthesis pathway in Drosha mutants (119). MiR-21 can activate the signal transduction downstream of TGF-β (120), while the combination of TGF-β and 1,25-dihydroxyvitamin D3 (VD3) can significantly increase the levels of 5-LOX in human monocytes (121). Accordingly, RSV can upregulate the level of leukotrienes by upregulating 5-LOX through the abnormal expression of miRNAs.

The activity of intracellular 5-LOX is strictly controlled by Ca2+, ATP, redox state, and phosphorylation (25, 122). However, nearly all of these factors can be affected by RSV (90, 123). Ca2+ regulates 5-LOX activity through the C2-like domain. Besides, Ca2+ increases the activation of MAPK and facilitates 5-LOX migrating to the nuclear membrane, which is necessary for leukotriene synthesis. By activating the p38 MAPK signal, RSV can directly activate 5-LOX by phosphorylation in monocytes (MM6) and polymorphonuclear leukocytes (PMNL) (124). In this process, miRNAs such as let-7 and miR-21 play a pivotal role (125, 126). Taken together, these examples reveal the possible mechanism of how miRNAs control the enzymatic activity of 5-LOX in RSV infection.

#### Expression of FLAP

Abnormal levels of miRNAs and FLAP have been well-documented in RSV infection. Besides, recent studies have found that miRNAs can suppress the formation of FLAP, one of the most critical factors of the 5-LOX catalysis function. MiR-135a and miR-199a-5p can target the 3′-UTR of mRNA to negatively regulate the expression of FLAP. Therefore, in hypoxia-induced endothelial cells, the downregulation of miR-135a and miR-199a-5p can increase the expression of FLAP (127). Another study has confirmed that hypermethylation of the miR-146a promoter leads to decreased expression of FLAP and leukotrienes in lung cancer cells, also by directly targeting the 3′-UTR of FLAP (64).

#### Expression of 12/15-LOX

The lipoxygenase pathway can also synthesize SPMs via 5-LOX and 12/15-LOX. SPMs are endogenous regulators of infection and inflammation, with a wide range of pro-inflammatory effects, such as inhibition of neutrophil and eosinophil chemotaxis, vascular adhesion, and transendothelial and transepithelial migration (65, 128–130). MiRNAs targeting 12/15-LOX may also contribute to the imbalanced inflammation in RSV infection. In a mouse hippocampal cell line, miR-181b directly binds to 12/15-LOX 3′-UTR, thereby negatively regulating 12/15-LOX expression (131). By inhibiting the expression of 12/15-LOX, miR-106a could reverse the effect that was induced by high glucose in the diabetic peripheral neuropathy mouse model (131). Moreover, overexpression of miR-219-2 in macrophages can increase 15-LOX and 12-LOX mRNA expression but decrease the expression of LTA_4_H (115). Recently, a study found that eicosapentaenoic acid's metabolism of 15-LOX promotes the expression of miR-101, thus inhibiting the COX2 pathway in colon cancer, which also implies the complicated links among miRNA, LOX pathway, and COX pathway (132).

### miRNAs and COX Pathway

Another vital way to increase leukotrienes is to inhibit the COX pathway. Prostaglandins (PGs), synthesized by AA through the COX pathway, are the most widely studied inflammation mediators by far. There is strong evidence that RSV infection can induce COX-2 with the concomitant production of PGs in AECs (37, 133). COX-2 is an inducible enzyme for PG synthesis, which is upregulated in inflammatory cells and thus induces an increase of PGs and inflammatory damage (134). Evidence for miRNAs controlling the expression of COX-2 is abundant. MiR-16 can complement with the AU-rich region of 3′UTR of COX-2, thus changing the stability of COX-2 mRNA (135). Overexpression of miR-216a-3p in human colorectal cancer cell lines can also inhibit the expression of COX-2(116). Similarly, both COX-2 and FLAP were downregulated by hypermethylation of the miR-146a promoter through directly interacting with the 3′UTR of the target mRNA (64). A study showed that NAD^+^-linked 15-hydroxyprostaglandin dehydrogenase (15-PGDH), which is part of the COX-2/PGE2 signaling pathway, was identified as a target of miR-21 in cholangiocarcinoma (71). In addition, miRNA can also inhibit the expression of COX-2 by reducing the level of the RNA-binding protein HUR in human hepatoma cells (68). Other miRNAs, such as miR-26, miR-30, miR-101, miR-137, miR-143, miR-144, miR-146a, miR-199a, and miR-216, are successively identified to modulate the COX pathway (24, 55, 66, 67, 69, 70), although their detailed mechanism has not been previously reported.

## Conclusion

The expression of miRNAs and leukotrienes changed dramatically after RSV infection. Overexpressed leukotrienes are closely related to AHR, mucous cell metaplasia, leukocyte aggregation, and airway barrier destruction following RSV infection. To explore the mechanism of action of miRNAs in RSV infection is important for developing strategies to restore the level of leukotrienes. MiRNAs can modulate the synthesis of leukotrienes by participating in posttranscriptional regulation of several key enzymes and associated activating proteins, which results in an imbalance between pro-inflammatory and pro-resolving mediators. Moreover, miRNAs can be secreted through the exosome and they have cellular specificity, so they can widely regulate the immune response after RSV infection. In summary, we described miRNAs that are abnormally expressed both in RSV infection and related to leukotriene synthesis, which may have important implications in the excessive inflammation of RSV infection and provide a potential therapeutic approach for the reasonable regulation of leukotriene expression after RSV infection.

## Author Contributions

ZL, PF, and DZ participated in writing the paper and collecting the articles and contributed for the linguistic revision of the manuscript. DZ guided the writing and editing of the article. MC and YX collected the references and revised the review. All authors contributed to the article and approved the submitted version.

## Conflict of Interest

The authors declare that the research was conducted in the absence of any commercial or financial relationships that could be construed as a potential conflict of interest.
